# Development and validation of a robust necroptosis related classifier for colon adenocarcinoma

**DOI:** 10.3389/fgene.2022.965799

**Published:** 2022-08-05

**Authors:** Jie Yang, Hua Chen, Yongqiang Wang, Jian Chen

**Affiliations:** Department of General Surgery, Medical College of Soochow University, Affiliated Kunshan Hospital of Jiangsu University, Kunshan, China

**Keywords:** colon adenecarcinoma, necroptosis, tumor microenvironment, risk model, genomic variations

## Abstract

**Background:** Necroptosis, a novel form of apoptosis, plays a crucial function in the progression of colon adenocarcinoma (COAD) and is expected to be triggered in cancer therapy for enhancing anti-tumor immunity. However, the function of necroptosis in tumors and its relationship with the tumor microenvironment (TME) remains largely unclear.

**Methods:** Necroptosis-related genes (NRGs) were collected from high-quality literature. Using The Cancer Genome Atlas (TCGA) (https://cancergenome.nih.gov) and the Gene Expression Omnibus (GEO) (www.ncbi.nlm.nih.gov/geo) meta-cohorts, a robust risk model was constructed to systematically examine the clinical value, functional status, the role of TME based on the risk model, as also the genomic variations.

**Results:** A risk model containing nine NRGs, including TNF receptor-associated factor (TRAF2), TNF receptor 1 associated via death domain (TRADD), ubiquitin carboxyl-terminal hydrolase 21 (USP21), TNF receptor superfamily, member 6 (FAS), tumor necrosis factor receptor superfamily 10B (TNFRSF10B), mitogen-activated protein kinase 8 (MAPK8), mixed lineage kinase domain-like (MLKL), TNF receptor-associated factor 5 (TRAF5), and recombinant receptor-interacting serine-threonine kinase 3 (RIPK3), was constructed. The risk model’s stability and accuracy were demonstrated in training, as also the validation cohorts; it was verified as an independent prognostic model for COAD. High-risk group patients developed “cold” tumors having active tumor proliferation and immunosuppression, while those in the low-risk group developed “hot” tumors with active immune and cell killing functions. Moreover, a higher number of copy number variations in the genome and fewer somatic mutations were found in high-risk group patients. Furthermore, higher sensitivity towards immunotherapy and chemotherapy was seen in patients of the low-risk group.

**Conclusion:** A reliable risk model based on NRGs to assess patient prognosis and guide clinical decision-making was constructed and validated. Our findings may contribute to the understanding of necroptosis and are expected to aid clinical management and guide precision treatment for patients with COAD.

## Introduction

Globally, colon adenocarcinoma (COAD) is the fourth most prevalent tumor with approximately 1.1 million new diagnoses and the fifth leading reason for cancer-associated deaths; 550,000 deaths were reported in 2018 alone (Bray et al., 2018). Given the advancements in precision medicine, substantial efforts have gone into refining personalized treatment and management of COAD. In general, strategies for treatment are largely dependent on validated prognostic features from previous studies. Moreover, tumor pathological staging remains a crucial determiner for the treatment and prognosis of colorectal cancer (CRC) (Sargent et al., 2010). However, the utility of the existing staging system is insufficient. Hence, there is a need to discover new biomarkers to predict patient prognoses and identify high-risk groups that are most likely to benefit from treatment. Recently, several developments have been in this field. For instance, Bao et al. report that microsatellite instability (MSI) is significantly associated with immunotherapeutic efficacy in COAD (Bao et al., 2020). Tumor mutation burden (TMB) has also been identified as a predictor of patient prognosis in several cancer types and is an emerging biomarker for assessing the sensitivity to immune checkpoint inhibitors (Chalmers et al., 2017; Samstein et al., 2019).

Necroptosis, a new kind of programmed cell death, was first reported in 2005 (Degterev et al., 2005). It is a genetically programmed, lysogenic apoptosis mechanism, that is regulated in a caspase-independent manner. It is an alternative mode of apoptosis that overcomes resistance and triggers to enhance anti-tumor immunity in cancer therapy (Gong et al., 2019; Tang et al., 2020). Activation of the protein kinases, including the recombinant receptor-interacting serine-threonine kinase 1 (RIPK1) and RIPK3, is involved in the onset of necroptosis, followed by phosphorylation of the executioner molecule, mixed lineage kinase domain-like (MLKL), further inducing rupture of the cell membrane (Chan, Luz, Moriwaki; Pasparakis and Vandenabeele, 2015; Sun et al., 2012). In cancer, necroptosis is a double-edged sword. If, on the one hand, apoptosis is not induced, necroptosis can provide an alternative, thereby eliciting a strong adaptive immune response and halting tumor progression. On the other hand, in a case where the recruited inflammatory response molecules promote tumorigenesis and metastasis, necroptosis may cause the tumor microenvironment (TME) to become immunosuppressive (Gong et al., 2019). Thus, there is a requirement to better construe the mechanisms underlying necroptosis and their physiological and pathological functions to address the queries on the value of necroptosis for patient prognoses, immune regulation, and therapy in cancer.

In the present study, 33 necroptosis-related genes (NRGs) were screened and analyzed for their patterns in COAD using multi-omic data. Further, 10 NRGs that were related to the prognosis were selected by Cox regression and modeled using an iterative least absolute shrinkage and selection operator (LASSO) regression analysis for COAD. Moreover, we systematically assessed the accuracy and stability of the prognostic model in both the training and the external validation cohorts and evaluated the prognostic model in detail for biological function, TME, and genomic variations. Finally, we determined the prognostic predictive ability of the model for chemotherapeutic and immunotherapeutic responses in COAD in clinical settings. A brief flow chart of this study was shown in Supplementary Figure S1.

## Methods

### Data extraction from online databases

The clinical information and corresponding data of transcriptomic RNA sequencing, HumanMethylation450 arrays, copy number variation (CNV), and Mutect2 mutation, of COAD patients, were downloaded from The Cancer Genome Atlas (TCGA) database (https://cancergenome.nih.gov/). Patients with incomplete clinical information were excluded. Thus, 432 COAD samples were used for subsequent analyses. The raw fragments per kilobase million (FPKM) data were normalized to transcript per million (TPM) and used as the training cohort. We also obtained three datasets from the Gene Expression Omnibus (GEO) database (https://www.ncbi.nlm.nih.gov/geo/) as follows: GSE14333 from GPL570, GSE17536 from GPL570, and GSE41258 from GPL96. The three GEO datasets comprising of 654 COAD patients consisted of the complete clinical information and batch effects were eliminated by the combat function of the R package, “sva” (Leek et al., 2012). These data were log2-transformed and used as a validation cohort. In addition, an immunotherapy cohort IMvigor210 was collected from http//research-pub.gene.com/IMvigor210CoreBiologies (Mariathasan et al., 2018). IMvigor210 contains 298 patients with uroepithelial carcinoma treated by anti PD-L1 therapy, and the source data were log2 normalized for assessing immunotherapeutic responses. Finally, 33 NRGs were included from previously published high-quality literature (Vandenabeele et al., 2010; Su et al., 2015; Gong et al., 2019; Yan et al., 2021), as listed in Supplementary Table S1.

### Construction and validation of an NRG-related risk model

Using the TCGA cohort, the model was trained. First, the prognosis-related NRGs were screened using univariate Cox regression, and, to avoid omission, those with *p* < 0.2 were used in subsequent analysis. Next, using a LASSO penalized Cox proportional risk model, the best prognostic model was identified after a 10-rule cross-validation to determine model stability. Assuming random sampling, 250 iterations were performed to identify the most stable prognostic model. Finally, the most stable prognostic model was selected to construct calculate the RiskScore as follows:

$$Risk\;Score=\sum^{\text{​}}iCoefficient\left(mRNA_{i}\right)\times Expression\left(mRNA_{i}\right)$$

The consistency index (c-index) was computed by the R package, “survcomp” to assess the predictive power of the RiskScore in the validation and training cohorts; a larger c-index indicated higher accuracy of the model (Schröder et al., 2011). In addition, correspondingly the patients were categorized into the high- and low-risk groups basis the median RiskScore.

### Functional enrichment analysis

Molecular markers for angiogenesis, myeloid inflammation, epithelial-mesenchymal transition (EMT), and other immune-related pathways were collected from previous studies (Ayers et al., 2017; Gibbons and Creighton, 2018; McDermott et al., 2018; Liang et al., 2020). Molecular markers for hypoxia were obtained from the Msigdb database (www.plob.org/tag/msigdb) (Liberzon et al., 2011). By single-sample gene set enrichment analysis (ssGSEA) using the R package, “gsva”, the pathway activities of the samples were assessed. Subsequently, the gene set enrichment analysis (GESA) was performed for the two risk groups to identify the subtypes that were significantly enriched in the KEGG pathways; the enrichment was considered significant at *p* < 0.05.

In addition, we collected the homologous recombination defect (HRD) scores, intratumor heterogeneity scores, and microsatellite instability (MSI) scores of the samples as described by Thorsson et al. (2018).

### Immune infiltration analysis

The relative infiltration activities of immune cell types in each sample were quantified using the “CIBERSORT” package in R and the “LM22″ background expression profile (Newman et al., 2015). The stromal and immune scores of the patients were computed using the ESTIMATE algorithm (Yoshihara et al., 2013).

### Landscape of genomic variation between the two groups

The total number of mutations in the samples was calculated to assess the differences in the mutation burdens between the high- and low-risk groups. Genes with a minimum number of mutations >30 were further identified using the “maftools” R package, and differences in mutation frequencies between two risk groups were contrasted by the chi-square test and visualized using the “maftools” package (Mayakonda et al., 2018). The CNV data were processed using Gistic (version: 2.0) to identify amplifications (value >0.3) and deletions (value < -0.3) at genetic loci using a threshold of 0.3; finally, the CNV landscape was visualized using the R package, Circos.

### Assessment of the clinical significance of the risk model

Using the pRRophetic package, we predicted the sensitivity of patients to four first-line COAD drugs (5-FU, cisplatin, paclitaxel, and doxorubicin) in the training and validation cohorts and estimated the half-maximal inhibitory concentration (IC50) values by ridge regression; the smaller was the IC50 value, the greater was the sensitivity to the drugs (Geeleher et al., 2014). The potential therapeutic targets were the differentially expressed genes (DEGs) in the two risk groups, and the CMap database (https://clue.io/) was used to identify the putative molecules which could target the DEGs (Subramanian et al., 2017). The top 150 upregulated and downregulated DEGs were queried for their corresponding possible small molecule compounds. Subsequently, an unsupervised subclass mapping algorithm (https://cloud.genepattern.org/gp/) and a webtool (http://tide.dfci.harvard.edu) (Jiang et al., 2018) and were used to assess the immunotherapeutic responses. Finally, the predictive utility of the RiskScore was verified in an immunotherapy cohort.

### Bioinformatics and statistical analyses

The R (version: 4.04) software was used for all statistical analyses and graph plotting. The Wilcoxon test was used to compute the differences between the two groups and compare them. To generate survival curves, the Kaplan-Meier plotter was used and statistically significant differences were assessed by the log-rank test. Time-dependent receiver operating characteristic curves (tROC) were plotted using the R package, “survivalROC”. Using the “survival” package in R, the univariate and multivariate Cox regression analyses were performed. Additionally, the “rms” package was used to construct the nomogram and plot the calibration curves. The decision curve analysis (DCA) was performed using the DCA package (Vickers et al., 2008). Unless stated otherwise, the two-tailed *p* < 0.05 was regarded as statistically significant.

## Results

### Landscape of genomic variations in NRGs in COAD patients

**FIGURE 1:**
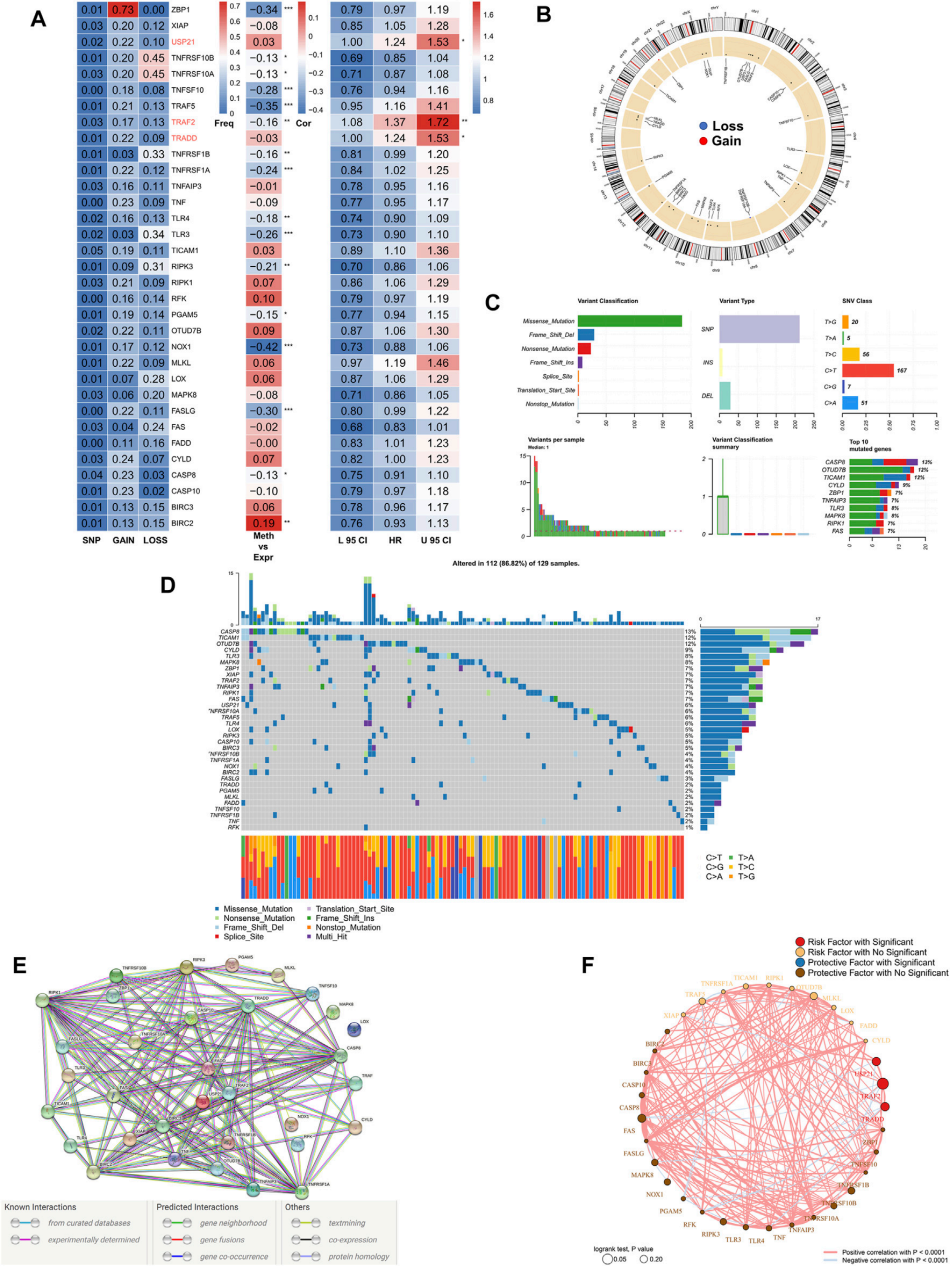
Genomic mapping of NRGs in COAD patients. **(A)**. Heat map showing genomic changes and hazard ratios of NRGs in TCGA-COAD cohort; from left to right: correlation between mutation and CNV frequencies for NRGs, modifications in DNA methylation and expression of NRGs, univariate Cox regression analysis showing risk ratios for NRGs; *p < 0.05, **p < 0.01, ***p < 0.001; **(B)**. Circle plot demonstrating CNV events in NRGs on chromosomes; **(C)**. Summary of CNV events in NRGs in TCGA-COAD cohort; **(D)**. Oncoplot showing the mutational mapping of NRGs; **(E)**. String PPI network of NRGs; **(F)**. Correlation network of NRGs.

First, we summarized the multi-omic profile of NRGs in TCGA-COAD patients (Figure 1A), whereby, a low frequency of mutations in NRGs but a wide range of CNVs, especially in tumor necrosis factor receptor superfamily 10B (TNFRSF10B), Z-DNA-binding protein 1 (ZBP1), and tumor necrosis factor receptor superfamily 10A (TNFRSF10A), were observed, which suggested that CNVs may exert a dominant effect in NRG regulation relative to gene mutations. In addition, DNA methylation also played a dominant role in the regulation of NRGs, especially in NADPH oxidase 1 (NOX1), ZBP1, TNFSF10, TNF receptor-associated factor 5 (TRAF5), and Fas ligand (FASLG). Moreover, three genes, including ubiquitin carboxyl-terminal hydrolase 21 (USP21), TRAF2, and TNF receptor 1 associated via death domain (TRADD), were identified as significant risk factors. Figure 1B demonstrates the CNV profile of NRGs on chromosomes. Next, we summarized the mutation profile of NRGs (Figure 1C) and observed that caspase 8 (CASP8), OTU domain-containing protein 7B (OTUD7B), and toll/interleukin-1 receptor domain-containing adapter molecule (TICAM1) were the three genes having the highest mutation frequency. Moreover, the most common mutation was missense; single nucleotide point mutation was the most common mutation type, very often resulting in the change in residue from cytosine to thymine. The waterfall diagram in Figure 1D shows the mutation profile of NRGs in patients. We then queried the NRGs for constructing the protein-protein interaction network using the STRING database (string-db.org) and (Figure 1E) obtained BIRC2 and BIRC3 as the hub genes at a confidence level of 0.9. Finally, we mapped the correlation network of NRGs, most of which were closely related, and thus, only the pairs with *p* < 0.0001 are shown (Figure 1F).

### Construction of the NRG-related risk model

**FIGURE 2:**
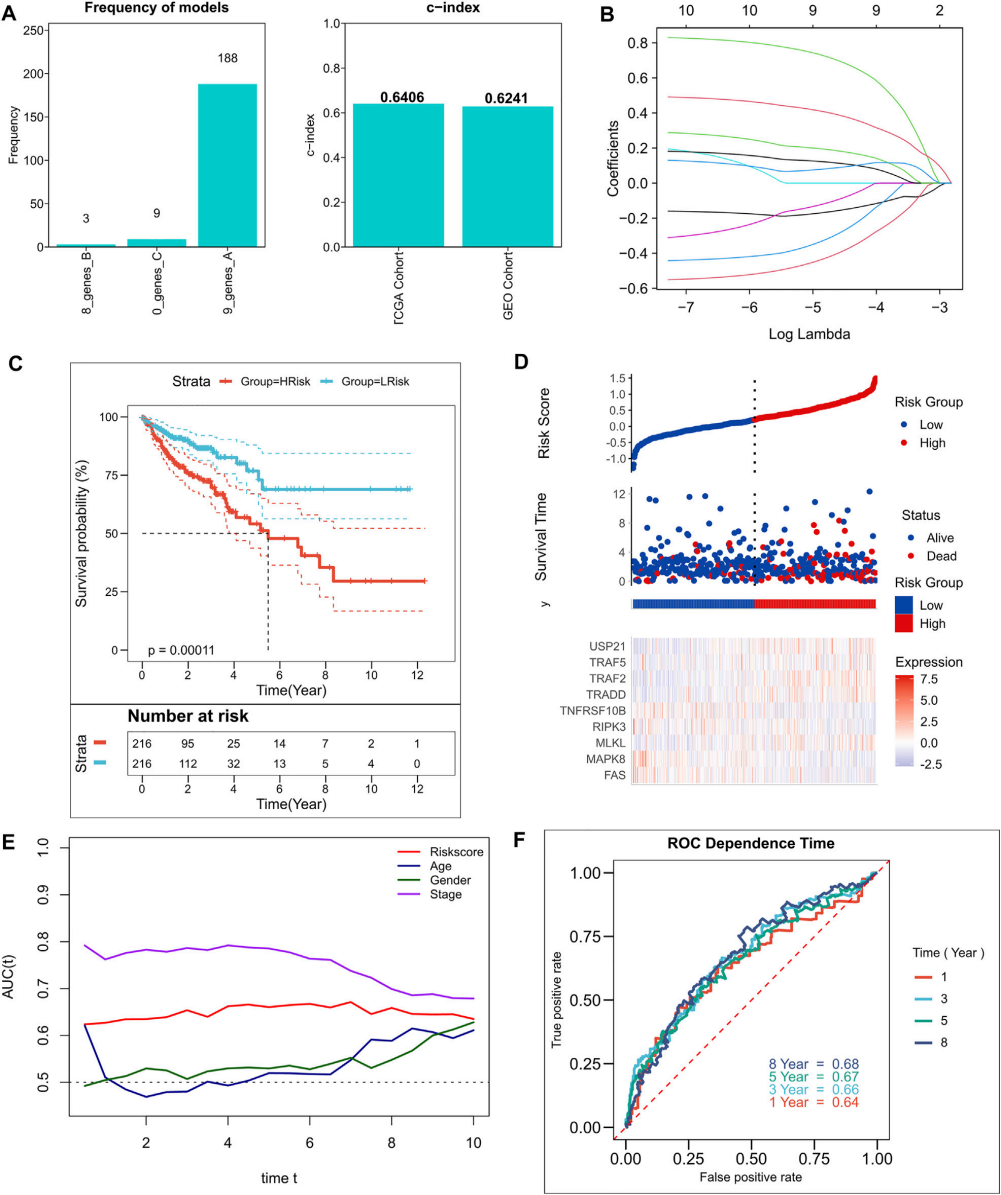
Construction of the NRGs-related risk model. **(A)**. Screening of the best LASSO model; left: frequency of different gene combinations in the LASSO Cox regression model, right: c-index of the best model in TCGA and GEO cohorts; **(B)**. LASSO Cox regression model to identify the top robust nine-gene marker having an optimal λ value of 0.007033; **(C)**. KM survival curves for the high- and low-risk groups in TCGA cohort. **(D)**. Survival status of patients and expression of model genes in TCGA cohort; **(E)**. tROC curves of risk models and clinical characteristics in TCGA cohort; **(F)**. 1-, 3-, 5-, and 8-years ROC curves for the RiskScore in TCGA cohort.

A total of 10 NRGs were identified as candidate genes in the model, including TRAF2, TRADD, USP21, FAS, MLKL, TNFRSF10B, MAPK8, TRAF5, RIPK3, and NOX1, with a threshold of *p* < 0.2, and the specific Cox results are listed in Supplementary Table S2. After 250 iterations in LASSO regression, we found that the model comprising nine genes, including TRAF2, TRADD, USP21, FAS, MLKL, TNFRSF10B, MAPK8, TRAF5, and RIPK3, was the most stable. This model had good accuracy in both the training and validation cohorts (TCGA: 0.6406; GEO: 0.6241) (Figure 2A). In addition, the model was constructed according to an optimal λ value of 0.007033 (Figure 2B), and the RiskScore was evaluated using the formula for RiskScore , with LASSO coefficients for the model genes listed in Supplementary Table S3. The patients were categorized based on the median RiskScore into high- and low-risk groups. In addition, survival analysis suggested that relative to those in the low-risk group, in the high-risk group, the patients had a significantly lower rate of survival (Figure 2C; *p* = 0.00011). Figure 1D shows the distribution of RiskScore and gene expression in TCGA cohort. Additionally, the tROC analysis showed that RiskScore was the best predictor in addition to staging (Figure 1E). Specifically, the 1-, 3-, 5-, and 8-years AUCs for the model were 0.64, 0.66, 0.67, and 0.68, respectively (Figure 1F). We also assessed the predictive utility of the model in the validation cohort, along with survival analysis, which suggested significantly worse survival in the high-risk group (Supplementary Figure S2A, *p* < 0.0001). Supplementary Figure S2B shows the model RiskScore distribution in the GEO cohort. The 1-, 3-, 5-, and 8-years AUCs were 0.63, 0.65, 0.66, and 0.66, respectively, for the model in the validation set (Supplementary Figure S2C).

### Assessment of the predictive independence of the risk model

**FIGURE 3:**
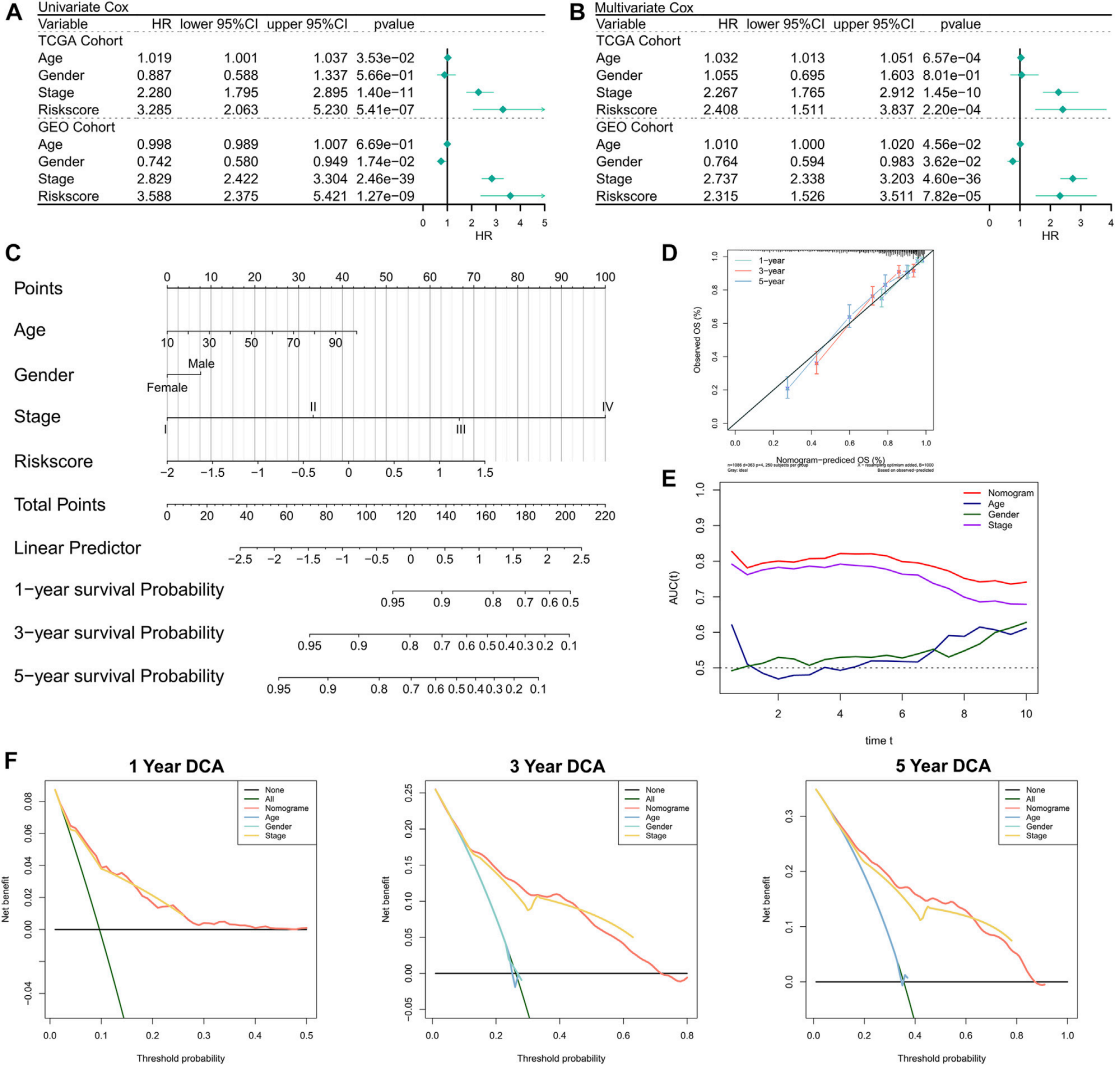
Validation of the NRG-related risk model. **(A)**. Univariate Cox regression analysis for OS in TCGA and GEO cohorts; **(B)**. Multivariate Cox regression analysis for OS in TCGA and GEO cohorts; **(C)**. Nomogram based on NRG-related risk model; **(D)**. Calibration curves for the nomogram; **(E)**. Clinical characteristics and tROC curves for the nomogram; **(F)**. 1-, 3-, and 5-years DCA curves for the nomogram.

First, the relationship between RiskScore and clinical parameters and patient prognoses was evaluated by univariate and multivariate Cox regression analyses. The results of the univariate Cox regression analysis suggested that the RiskScore (hazard ratio [HR] = 3.285, *p* < 0.001), TNM stage (HR = 2.280, *p* < 0.001), and age (HR = 1.019, *p* = 0.0353) in the training cohort were significantly associated with patient prognosis (Figure 3A); RiskScore (HR = 3.588, *p* < 0.001), TNM stage (HR = 2.829, *p* < 0.001), and gender (Female versus male; HR = 0.742, *p* = 0.0174) in the validation cohort were significantly associated with patient prognosis (Figure 3A). The results of the multivariate Cox regression analysis suggested that after correction for clinical characteristics, the RiskScore remained an independent prognostic factor for the overall survival (OS) of patients (TCGA: HR = 2.408, *p* < 0.001; GEO: HR = 2.315, *p* < 0.001) (Figure 3B). Hence, the RiskScore could serve as a prognostic marker for OS in COAD patients. In addition, we constructed a nomogram to better quantify the risk assessment of COAD patients (Figure 3C). The correction curve of the nomogram indicated good stability Figure 3Dand accuracy (Figure 3D). Moreover, the tROC analysis showed that the nomogram was the best predictor relative to the clinical characteristics (Figure 3E). We then performed a DCA for the nomogram to assess its decision benefit and the results showed that the nomogram was useful for risk assessment of patients with COAD at 1-, 3-, and 5-years (Figure 3G).

### Functional enrichment analysis of the risk model

**FIGURE 4:**
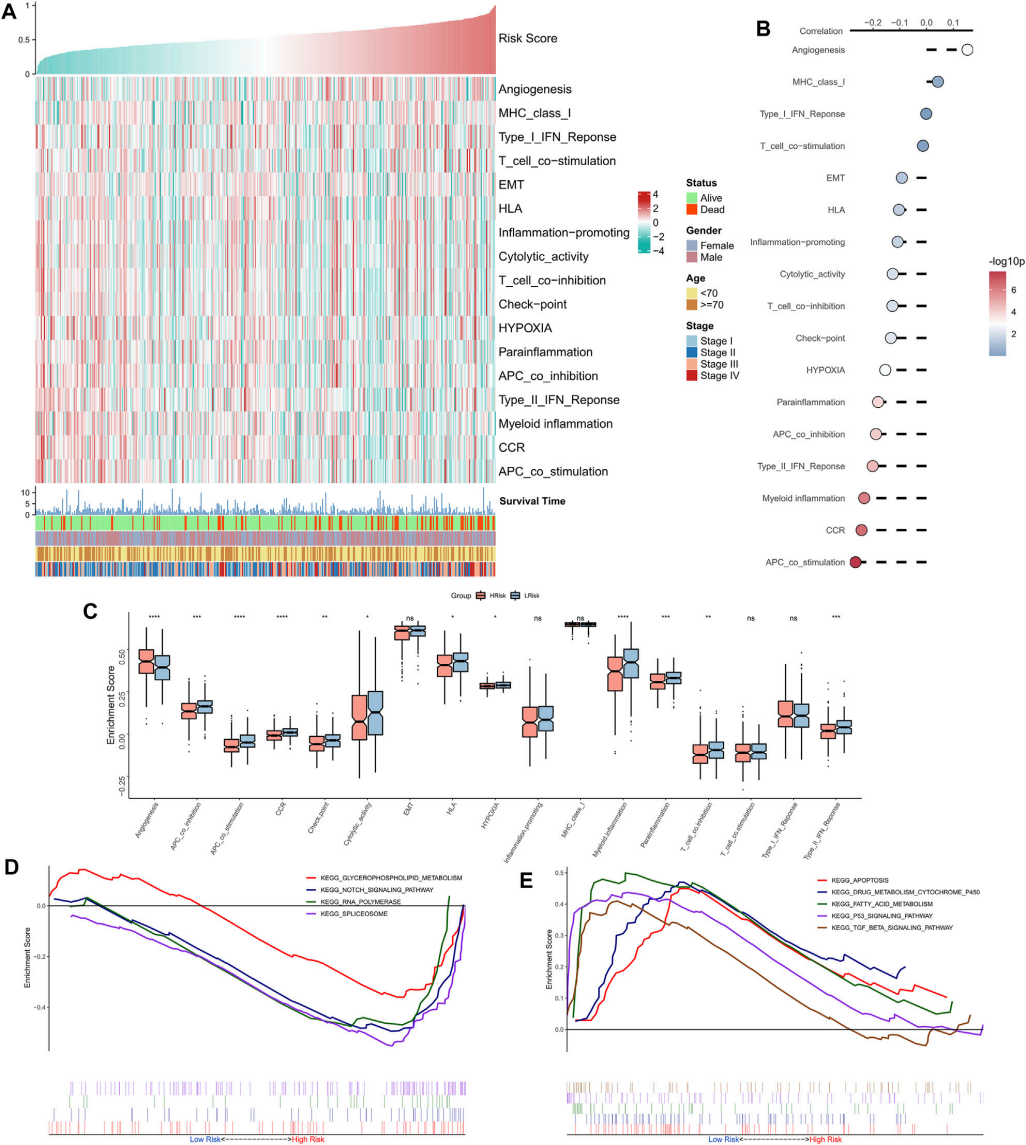
Functional analysis of the NRG-related risk model. **(A)**. Heat map showing the correlation between RiskScore, activities of biological pathways, and clinical characteristics; **(B)**. Correlation analysis between RiskScore and biological pathways; **(C)**. Box plots showing the differences in activities of the biological pathways between the high-risk and low-risk groups; **(D)**. GSEA enrichment plot showing the four pathways of interest in the high-risk group; **(E)**. GSEA enrichment plot showing the 5 pathways of interest in the low-risk group.

The correlation between RiskScore and some typical biological pathways was assessed. The heat map shows the relationship between RiskScore, activities of the biological pathways, and clinical characteristics (Figure 4A). The RiskScore showed a positive association with angiogenesis and a negative association with hypoxia and certain immune-related pathways (e.g., APC co-stimulation, CCR, Type II interferon response, and myeloid immunity) (Figure 4B). Consistently, we observed that angiogenesis was markedly elevated in the high-risk group, whereas hypoxia and certain immune-related pathways (e.g., APC co-stimulation, myeloid immunity, CCR, and Type II interferon response) were substantially upregulated in the low-risk group (Figure 4C). GSEA showed that the RNA polymerase and spliceosome signaling pathways were markedly enhanced in the high-risk group, whereas P53, apoptosis, and transforming growth factor-beta signaling pathways were substantially upregulated in the low-risk group (Figure 4D). In summary, these results suggested active cell proliferation and tumor angiogenesis in the high-risk group. Immune hyperfunction characterized the low-risk group.

### Immune landscape of the risk model

**FIGURE 5:**
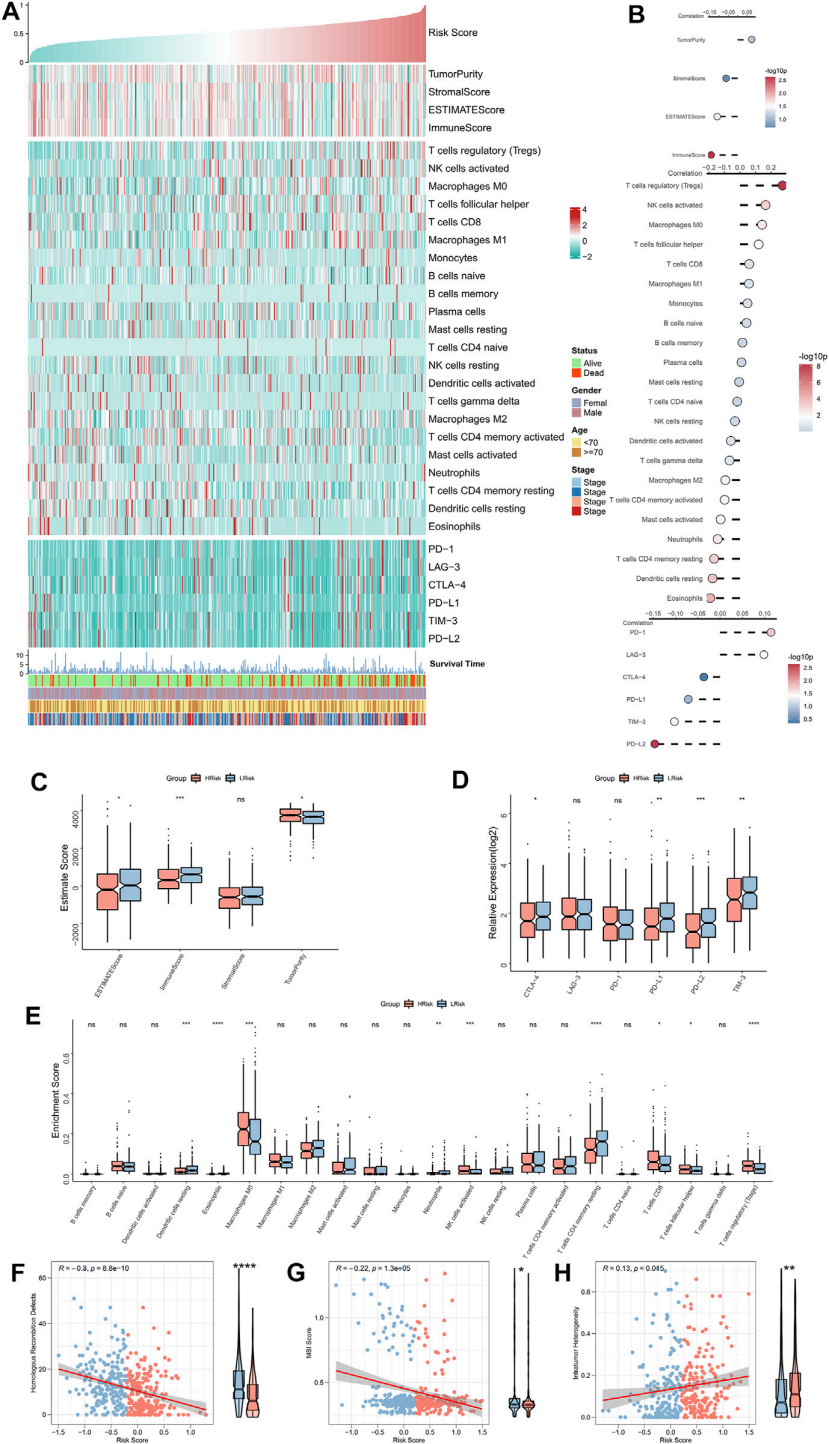
Immune landscape of the NRG-related risk model. **(A)**. Heat map showing the correlation between RiskScore, EstimateScore, the abundance of immune cell infiltration, immune checkpoint expression, and clinical characteristics; **(B)**. From top to bottom: correlation analysis between RiskScore and EstimateScore, immune cell infiltration abundance, and immune checkpoint expression; **(C)**. Box plot showing the differences in the abundances of immune cell infiltrations between the high-risk and low-risk groups; **(D)**. Box plot showing the differences in EsimateScore between the high-risk and low-risk groups; **(E)**. Box plot showing the differences in immune checkpoint expressions between the high-risk and low-risk groups; **(F)**. Correlation between RiskScore and HRD scores; **(G)**. Correlation between RiskScore and MSI scores; **(H)**. Correlation between RiskScore and intratumor heterogeneity scores. *p < 0.05; **p < 0.01; ***p < 0.001.

The correlation between RiskScore and the immune landscape was assessed in further detail. The heat map shows the association between RiskScore, EstimateScore, the abundance of immune infiltration cell types, typical immune checkpoints (including CTLA-4, TIM-3, PD-1, LAG-3, PD-L1, and PD-L2), and clinical characteristics (Figure 5A). The corresponding correlation analysis is shown on the right of the heat map (Figure 5B). The immune score was significantly positively correlated with the Riskscore. Moreover, the box plot indicated that the immune score was markedly up-regulated in the low-risk group, while the tumor purity significantly ascended in the high-risk group (Figure 5C). Although the correlation analysis showed a significant positive association of LAG-3 and PD-1 with RiskScore, the box plot indicated that LAG-3 and PD-1 were not significantly elevated in the high-risk group, however, the remaining four immune checkpoints were markedly elevated in the low-risk group (Figure 5D). The box plot shows an enhanced abundance of follicular helper T cells, Tregs, CD8 T cells, M0 macrophages, and activated NKT cells, in the high-risk group, while in the low-risk group, resting CD4 memory T cells, acidic granulocytes, neutrophils, and resting dendritic cells, were elevated (Figure 5E). Although the high-risk group appears to have increased cell-killing activity, the significantly higher Treg infiltration herein can suppress the immune responses (Tanaka and Sakaguchi, 2017; Knochelmann et al., 2018). These findings further suggested that the immunological function was active in the low-risk group but was suppressed in the high-risk group. We then assessed two indicators associated with tumor-specific antigens, including HRD and MSI scores. The results showed that RiskScore was significantly negatively related to the HRD and MSI scores and that both of these were significantly high in the low-risk group (Figures 5F,G), which suggested that there were greater chromosomal instability alterations and tumor-specific neoantigens in the low-risk group (Ganesh et al., 2019; Eso et al., 2020; Shi et al., 2021). Finally, a significantly positive association was found between intratumor heterogeneity score and RiskScore; the former was also markedly greater in the high-risk group (Figure 5H), hinting at the tumor complexity and the tendency for malignancy in the high-risk group.

### Correlation between riskscore and genomic variation

**FIGURE 6:**
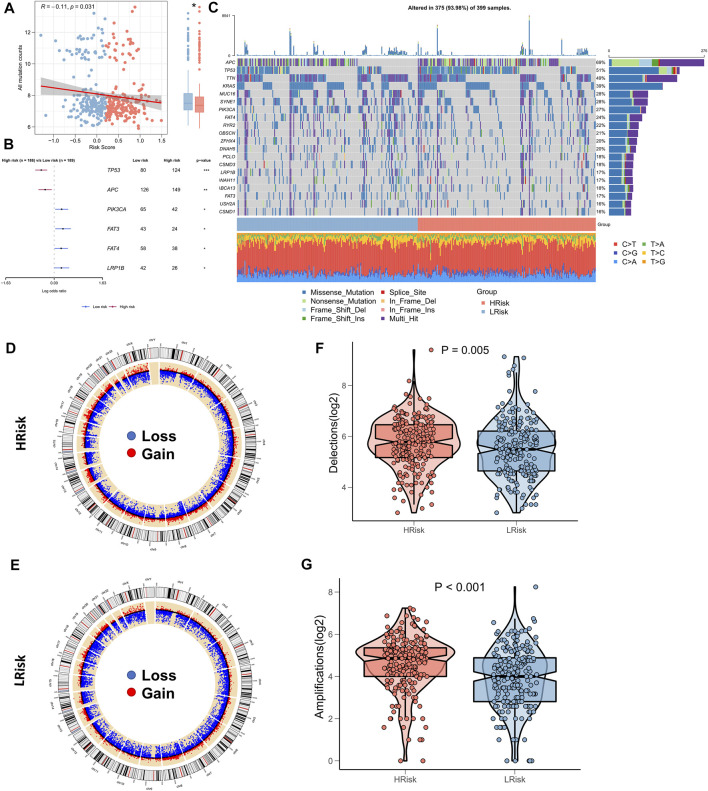
Landscape of genomic variations for NRG-related risk model. **(A)** Correlation between RiskScore and TMB; **(B)**. Forest plot showing genes with significant mutational differences between the high-risk and low-risk groups; **(C)**. Oncoplot showing significantly mutated genes between the high-risk and low-risk groups; **(D)**. Circle plot showing the CNV landscape in the high-risk group; **(E)**. Circle plot showing the CNV landscape in the low-risk group; **(F)**. Box plot showing the differences in the number of chromosomal deletions between the high-risk and low-risk groups; **(G)**. Box plots showing the differences in the number of chromosome amplifications between the high-risk and low-risk groups.

Several recent reports indicate that TMB correlates with immunotherapeutic responses, as somatic mutations may generate more potentially mutation-derived antigens that are recognized by the immune system, and such a recognition of the antigen-containing mutant peptides by the immune system can activate immune functions and enhance anti-tumor immunity (Matsushita et al., 2012; Rizvi et al., 2015; Chan et al., 2019). Given the clinical significance of TMB, the correlation between TMB and RiskScore was examined. A significantly negative association between TMB and RiskScore (correlation = -0.11, *p* = 0.031) was found; the TMB in the low-risk group was significantly high (Figure 6A). We further compared the mutation frequencies of the frequently mutated genes in the two groups. The Forestplot showed that TP53 and APC were significantly more commonly mutated in the high-risk group, whereas PIK3CA, FAT3, FAT4, and LRP1B were more commonly mutated in the low-risk group (Figure 6B). The landscape of the top 20 driving mutant genes was shown in Figure 6C. As CNV causes chromosomal variations too, we further evaluated the correlation between the RiskScore and CNV. Higher CNV events were observed in the high-risk group (Figure 6D) relative to the low-risk group (Figure 6E). The box plots showed a significant increase in both deletion and amplification events in the high-risk group (Figures 6F,G).

### Role of the risk model in guiding clinical decision-making

**FIGURE 7:**
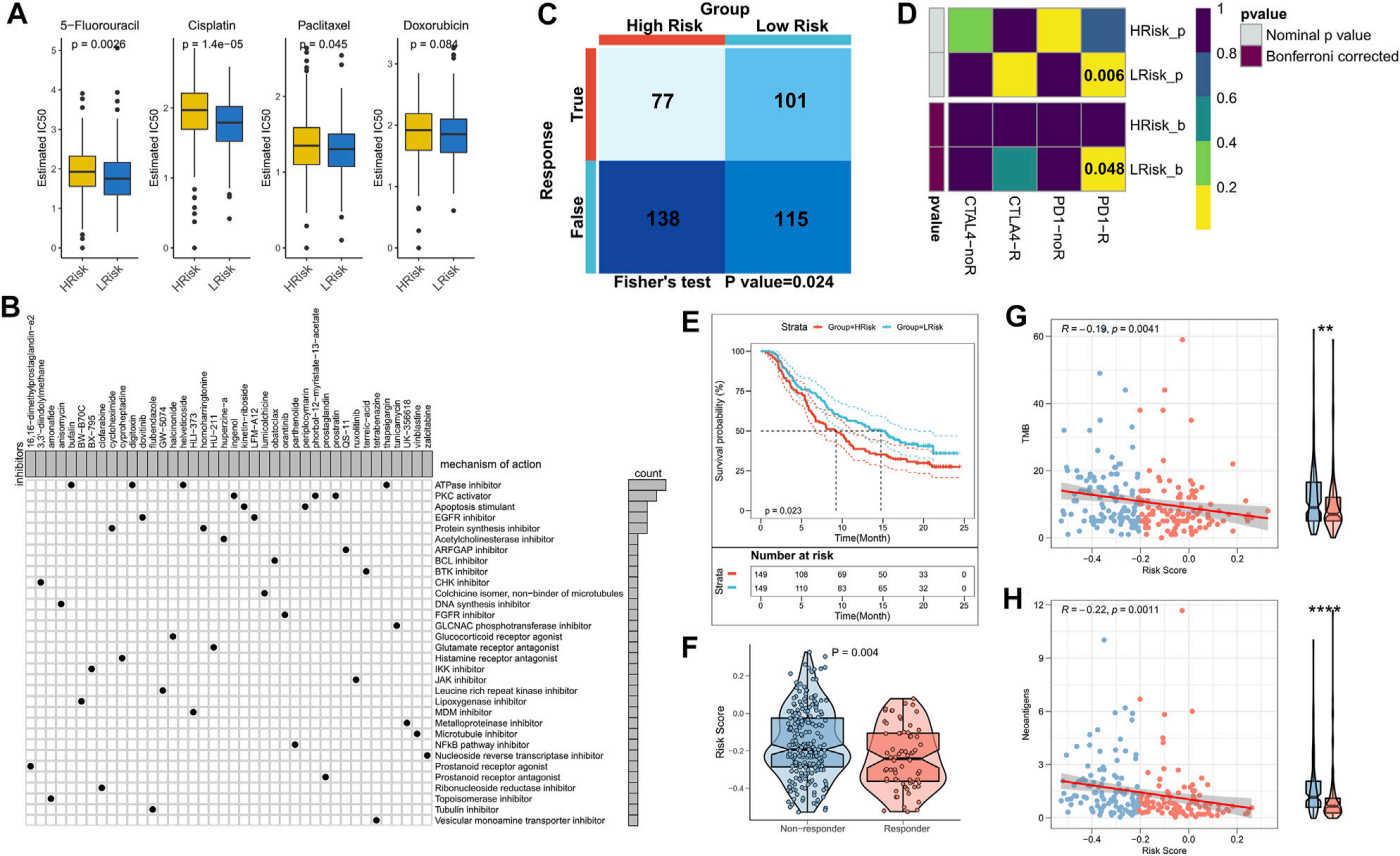
Role of the NRG-related risk model in guiding clinical treatment and decision-making. **(A)**. Box plot showing predicted IC50 values for four commonly used drugs in the high-risk and low-risk groups; **(B)**. Oncoplot showing the small molecule compounds, wherein the horizontal axis represents the name of the small molecule inhibitor and the vertical axis represents the biological pathway targeted by the corresponding small molecule inhibitor; **(C)**. TIDE algorithm for predicting responses to immunotherapy between the high-risk and low-risk groups; **(D)**. Subclass mapping for predicting sensitivity to PD1 and CTLA4 treatment in patients belonging to the high-risk and low-risk groups; **(E)**. KM survival curves for the high-risk and low-risk groups in the IMvigor 210 cohort; **(F)**. Box plot showing the differences in RiskScore between patients in the treatment-responsive and non-responsive groups of the IMvigor 210 cohort; **(G)**. Correlation between RiskScore and TMB in the IMvigor210 cohort; **(H)**. Correlation between RiskScore and neoantigens in the IMvigor 210 cohort.

The sensitivity of the patients to COAD chemotherapeutic agents in training and validation cohorts was evaluated and the findings suggested that patients in the low-risk group of TCGA cohort were more sensitive to 5-FU, paclitaxel, and cisplatin (Figure 7A). Patients in the low-risk group of the validation cohort were more sensitive to 5-FU, cisplatin, and Doxorubicin (Supplementary Figure S3A). Overall, a higher sensitivity to chemotherapy was seen in the patients in the low-risk group. Next, the DEGs were queried in the Clue database for identifying small molecule drugs, and as shown in the waterfall diagram, 41 potential small molecule drugs, and their corresponding target 33 biological pathways were identified (Figure 7B). Following previous results that suggest the potential of risk models for guiding immunotherapy, we assessed the patient response rates to immunotherapy using the Tumor Immune Dysfunction and Exclusion (TIDE) algorithm (tide.nki.nl), which showed that those in the low-risk group in TCGA cohort had a greater chance of responding to immunotherapy (*p* = 0.024, Figure 7C). Similar results were found in the GEO cohort, wherein patients in the low-risk group had a greater probability of responding to immunotherapy (*p* = 0.005, Supplementary Figure S3B). Subsequently, the subclass mapping results suggested that patients in the low-risk group of both TCGA and GEO cohorts were more sensitive to anti-PD1 therapy (TCGA: false discovery rate [FDR] = 0.048, GEO: FDR = 0.035) (Figure 7D; Supplementary Figure S3C). Finally, we computed the RiskScore in a well-established immunotherapy cohort, which showed significantly worse survival in the high-risk group (*p* = 0.023, Figure 7E). The RiskScore was significantly higher in patients who did not respond to immunotherapy (Figure 7F). We then evaluated the relationship of TMB and neoantigens with RiskScore in the immunotherapy cohort, which showed a negative correlation of the RiskScore with TMB and neoantigen count; both TMB and neoantigen count were significantly elevated in the low-risk group (Figures 7G,H). These results confirmed that this risk model may be a powerful tool for guiding immunotherapy selection for patients with COAD.

## Discussion

In the present study, based on NRGs, a prognostic model for COAD patients was constructed using a robust LASSO algorithm, followed by an in-depth analysis of the prognostic model for function, immune microenvironment, genomic variations, and clinical therapies. We examined the putative biological functions of NRGs in COAD patients. We confirmed the suitability and accuracy of the constructed prognostic model for predicting survival in COAD patients in both cohorts. Functional analysis suggested that patients in the high-risk group had active cell proliferation and tumor angiogenesis, while immune hyperfunction was a characteristic of the low-risk group. Additionally, immune microenvironment analysis also demonstrated better immunogenicity in COAD patients with low RiskScores. Analysis of genomic variations suggested that TP53 and APC had higher mutation counts in the high-risk group. Moreover, chromosomal amplification and deletion events were also significantly higher in the high-risk group. For clinical settings, we determined that in the low-risk group, the patients were more sensitive to COAD chemotherapeutic agents. Finally, we predicted better immunotherapeutic response in COAD patients with low RiskScores using TIDE and subclass mapping algorithms; these were validated in an external immunotherapy cohort.

Apoptosis is strongly associated with cancer progression, metastasis, and treatment response. Inhibiting apoptosis enhances tumor metastasis and resistance of malignant cells against chemotherapy (Su et al., 2015; Strasser and Vaux, 2020). Ferroptosis, pyroptosis, and necroptosis are emerging forms of apoptosis. As most tumors are innately resistant to apoptosis, the induction of apoptosis mechanisms is emerging as a new strategy for cancer treatment (Tang et al., 2020). The predictive values of pyroptosis and ferroptosis for the prognoses of COAD patients have been demonstrated (Nie et al., 2021; Zhuang et al., 2021). In the present study, wherein, necroptosis as the non-apoptotic program cell death mechanism was focused on, we found that USP21, TRAF2, and TRADD were significant risk factors for COAD. Moreover, the NRG-based risk model showed excellent predictive abilities in both the training and external validation cohorts; a markedly low survival rate was found in the high-risk group.

The association of the risk model and biological pathways was analyzed to examine the functional biology underlying the survival differences. We found that angiogenic activity was significantly higher in the high-risk group. Previous studies report that active angiogenesis is critical for tumor growth and metastasis and is substantially associated with suppression of immune function; inhibition of angiogenesis is also a promising therapeutic strategy for impeding tumor growth (Sharma et al., 2001; Motz and Coukos, 2011; Welti et al., 2013). However, immune-related pathways, such as cell killing, CCR, antigen presentation, interferon response, and myeloid immunity were found to be more active in the low-risk group, which suggested that antigen presentation, anti-tumor immunity, and cell killing were more potent in the low-risk group (Luo et al., 2017; McGranahan et al., 2017; Miar et al., 2020). The above findings suggested that tumor growth and treatment resistance in the high-risk group resulted in significantly poorer survival of patients in the high-risk group; while the low-risk group exhibited strong anti-tumor immunity.

As TME and immune activity are strongly associated with cancer treatment and prognosis (Bruni et al., 2020; Riera-Domingo et al., 2020), we assessed the differences in TME and immune activity between the risk groups. Notably, the low-risk group had higher immune scores and immune checkpoint activity, which suggested that the low-risk group was relatively immunocompetent. Although patients in the high-risk group appeared to have higher cell-killing activity, such as by NK cells and CD8 T cells, significantly elevated Tregs could suppress the immune responses in the high-risk group, thereby leading to immune escape (Tanaka and Sakaguchi, 2017; Knochelmann et al., 2018). In contrast, dendritic cells, acidic granulocytes, resting CD4 memory T cells, and neutrophils showed elevated abundance in the low-risk group, which suggested that patients in the low-risk group had a greater capacity for antigen-presentation and intrinsic immunity (Wculek et al., 2020). The above findings suggested that in the high-risk group, the patients developed immunosuppressed ‘cold’ tumors with a weaker anti-tumor response, leading to poorer prognoses. In contrast, patients in the low-risk group developed immunocompromised ‘hot’ tumors, leading to better prognoses.

TMB is a biomarker of immunotherapeutic response. In general, higher TMB predicts greater benefit from immunotherapy, but there is variability in its prognostic role in different tumors (Chalmers et al., 2017; Liu et al., 2019). Higher TMB in the low-risk group was found in the present study. Additionally, the mutation frequencies of TP53 and APC were markedly greater in the high-risk group, whereas those of PIK3CA, FAT3, FAT4, and LRP1B were higher in the low-risk group. Considering that the low-risk group has a robust immune function, indeed, patients in the low-risk group seemed to benefit more from immunotherapy. We also analyzed the pattern of CNVs in TCGA-COAD cohort and found that patients in the high-risk group had greater chromosomal amplification and deletion events. Studies show that somatic structural rearrangements in chromosomes actively drive oncogenesis and lead to greater tumor heterogeneity and chemoresistance (Stephens et al., 2009; Stephens et al., 2011; Waddell et al., 2015). These results suggested that patients in the low-risk group may be more sensitive to immunotherapy and chemotherapy than those in the high-risk group.

Many studies have shown that bioinformatics has amazing prospects in dealing with genomic variation, TME, and precision therapy (Jiang et al., 2020; Wang et al., 2021; Jiang et al., 2022; Yu et al., 2022). As previous results strongly suggested higher sensitivity to treatment among patients in the low-risk group, we finally analyzed the sensitivity of COAD patients in both groups towards chemotherapy and immunotherapy. In both cohorts, we confirmed that patients in the low-risk group were more sensitive to cisplatin and 5-FU. In addition, TIDE and subclass mapping algorithms also predicted that patients in the low-risk group were more sensitive to PD1 immunotherapy. We confirmed greater sensitivity to PD-L1 treatment and a longer survival time in the low-risk group using an external immunotherapy cohort. This may be because these patients had elevated TMB and neoantigen counts. In conclusion, these results affirmed that the risk model constructed in this study was a powerful tool and may have implications in guiding the treatment of patients with COAD.

There are certain limitations to the present study. First, due to the paucity of data, we only considered inter-patient heterogeneity and not intratumoral heterogeneity. Second, although we have used certain algorithms to determine the accuracy of this risk model in predicting the sensitivity of patients to chemotherapy and immunotherapy, further validation in prospective cohort studies and clinical data is required. Finally, *in vitro* and *in vivo* experiments are necessary to confirm the specific biological functions of NRGs in COAD.

In summary, this study pioneered the construction of the NRG-based risk model and identified high- and low-risk patients, showing heterogeneity in functional status, immune microenvironment, genomic variants, and clinical outcomes. In addition, the constructed risk model can be applied to predict the sensitivity of COAD patients toward immunotherapy and chemotherapy. Overall, these results are expected to advance the understanding of necroptosis, clinical management, and precise treatment options for patients with COAD.

## Data Availability

The original contributions presented in the study are included in the article/Supplementary Material, further inquiries can be directed to the corresponding author.

## Supplementary material

Supplementary Figure S1 The brief workflow of this study.

Supplementary Figure S2 The NRGs related risk model in GEO cohort. **(A)**.KM survival curves for the high- and low-risk groups in the GEO cohort. **(B)**.Survival status of patients and expression of marker genes in GEO cohort; **(C)**.1-,3-,5-,and8-year ROC curves for the RiskScore in GEO cohort.

Supplementary Figure S3 Validation of the treatment decisions in GEO cohort. **(A)** .Boxplot showing predicted IC50 values of four commonly used drugs between the two risk groups in the GEO cohort; **(B)**.TIDE algorithm for predicting immunotherapeutic responses of two risk groups in the GEO cohort; **(C)**.Subclass mapping for predicting sensitivity to anti-PD1 and anti-CTLA4 treatment in patients belonging to the high-risk and low-risk groups.
