# Supplementary figures and images for: Development and validation of a robust necroptosis related classifier for colon adenocarcinoma
Source: Front Genet. 2022 Aug 5;13:965799. doi: 10.3389/fgene.2022.965799 (PMC9389450; doi:10.3389/fgene.2022.965799)

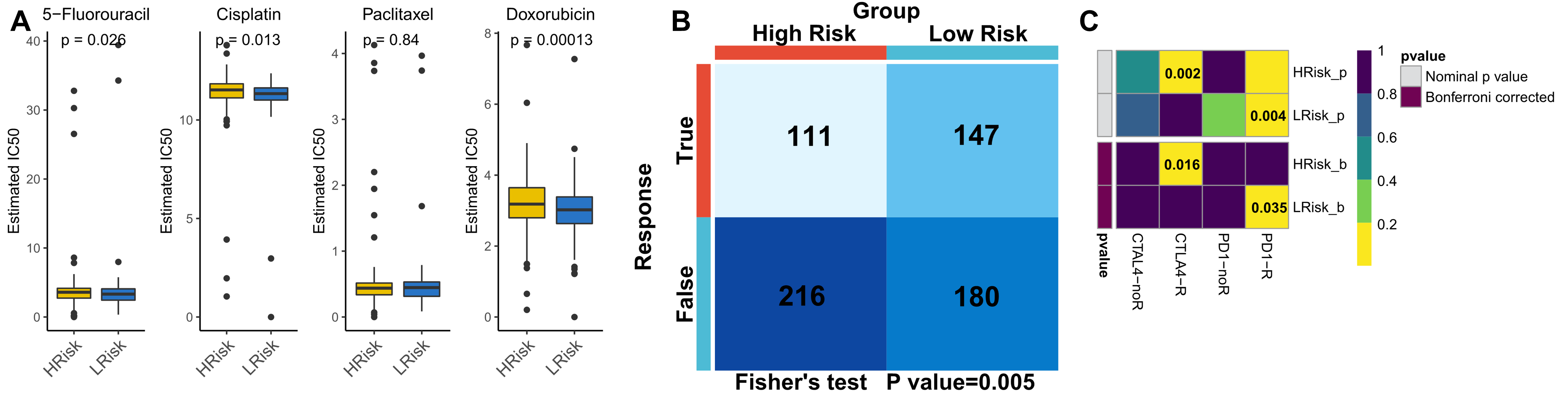

Supplement: Supplementary file 3 [file Image3.tif]

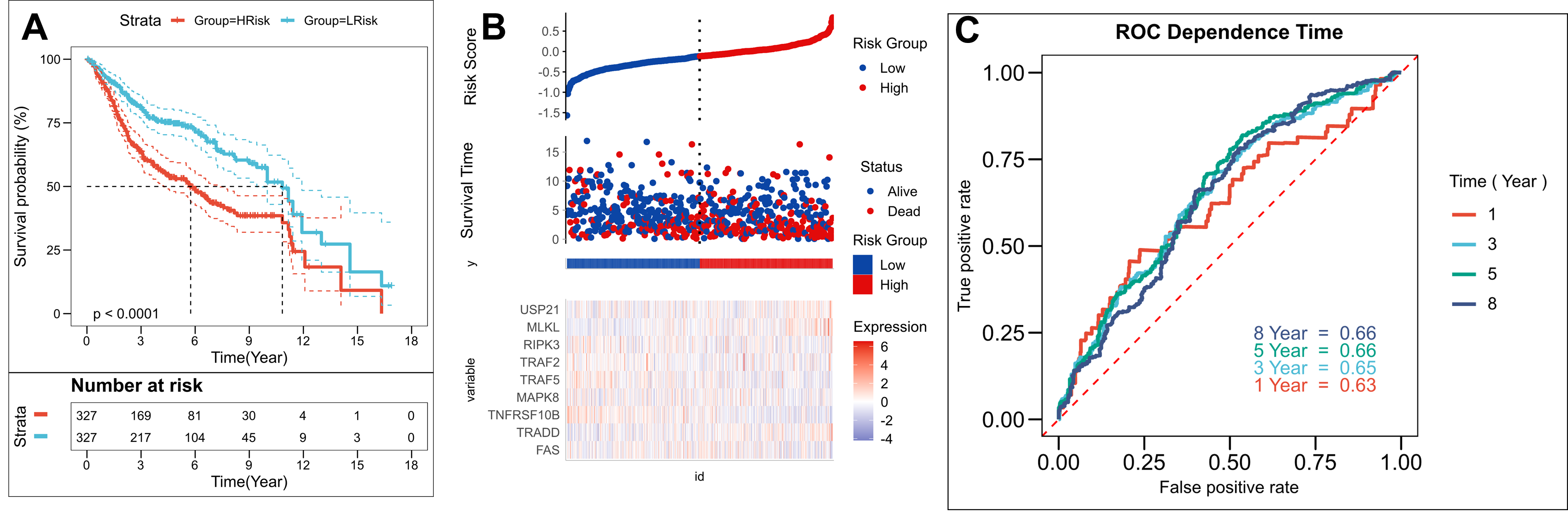

Supplement: Supplementary file 4 [file Image2.tif]

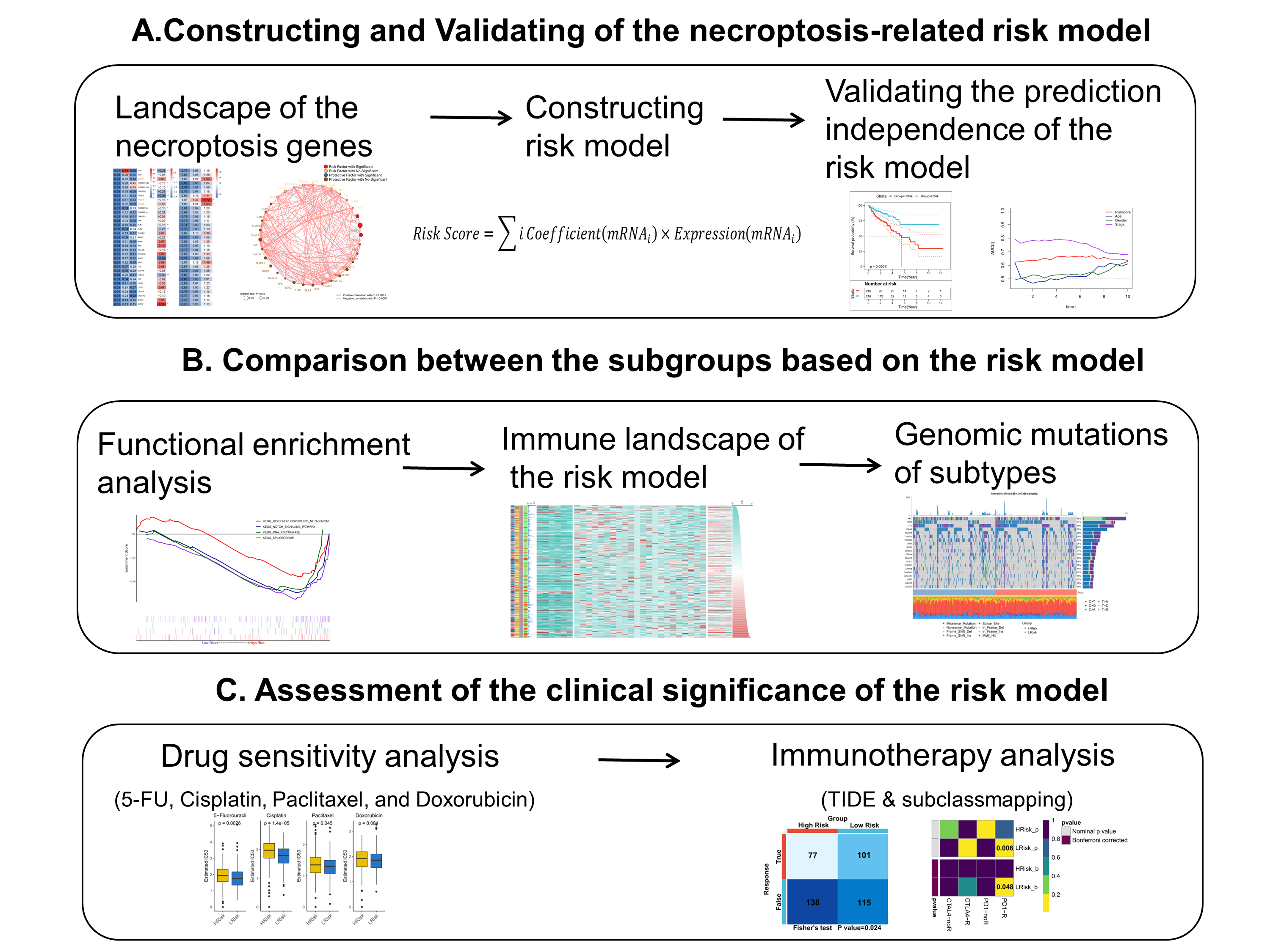

Supplement: Supplementary file 6 [file Image1.tif]
